# Serologic Evidence of the Geographic Distribution of Bacterial Zoonotic Agents in Kenya, 2007

**DOI:** 10.4269/ajtmh.15-0320

**Published:** 2016-01-06

**Authors:** Victor O. Omballa, Raymond N. Musyoka, Amy Y. Vittor, Kabura B. Wamburu, Cyrus M. Wachira, Lilian W. Waiboci, Mamo U. Abudo, Bonventure W. Juma, Andrea A. Kim, Joel M. Montgomery, Robert F. Breiman, Barry S. Fields

**Affiliations:** Center for Global Health Research, Kenya Medical Research Institute, Nairobi, Kenya; University of Tampere, Tampere, Finland; Africa Refugee Health Program, Division of Global Health Protection, Centers for Disease Control and Prevention, Nairobi, Kenya; Department of Medicine, University of Florida, Gainesville, Florida; University of Nairobi, Nairobi, Kenya; Kenya Ministry of Public Health and Sanitation, Nairobi, Kenya; Diagnostics and Laboratory Systems Program, Division of Global Health Protection, Centers for Disease Control and Prevention, Nairobi, Kenya; Division of Global HIV/AIDS, Surveillance and Epidemiology, Centers for Disease Control and Prevention, Nairobi, Kenya; Global Disease Detection Branch, Division of Global Health Protection, Centers for Disease Control and Prevention, Nairobi, Kenya; Emory University, Atlanta, Georgia

## Abstract

Diseases of zoonotic origin contribute to the burden of febrile illnesses in developing countries. We evaluated serologic evidence of exposure to *Bacillus anthracis*, *Brucella* spp., spotted fever group rickettsioses (SFGR), and typhus group rickettsioses (TGR) from samples of persons aged 15–64 years collected during a nationwide human immunodeficiency virus (HIV) serosurvey conducted in 2007 in Kenya. The seropositivity observed for pathogens was *B. anthracis* 11.3%, *Brucella* spp. 3.0%, SFGR 23.3%, and TGR 0.6%. On univariate analysis, seropositivity for each pathogen was significantly associated with the following risk factors: *B. anthracis* with province of residence; *Brucella* spp. with sex, education level, and wealth; SFGR with age, education level, wealth, and province of residence; and TGR with province of residence. On multivariate analysis, seropositivity remained significantly associated with wealth and province for *B. anthracis*; with sex and age for *Brucella* spp; and with sex, education level, and province of residence for SFGR whereas TGR had no significance. High IgG seropositivity to these zoonotic pathogens (especially, *B. anthracis* and SFGR) suggests substantial exposure. These pathogens should be considered in the differential diagnosis of febrile illness in Kenya.

## Introduction

Bacterial zoonotic pathogens are enzootic and endemic in many regions of the world, resulting in high morbidity and mortality, economic burden, and burden on the health-care system.^1^–^3^ In sub-Saharan Africa (SSA), bacterial zoonotic diseases are often undiagnosed because of limited diagnostic capacities and nonspecific clinical presentations, low index of clinical suspicion, leading to inappropriate treatment (especially in malaria-endemic areas), which often results in poor prognosis.^4^,^5^ Nonspecific clinical symptoms for bacterial zoonotic pathogens include fever, joint pains, and headache.^4^ Other factors that contribute to the risk of bacterial zoonotic diseases in SSA include human and livestock or wildlife interactions such as eating undercooked meat and drinking unpasteurized milk from infected animals and arthropod contact. Lack of recognition and treatment is partially attributable to infections occurring in remote areas where patients have limited access to health care and may be seen by providers with limited knowledge of and access to diagnostic tests for zoonotic illnesses and where there may be minimal surveillance.^1^,^3^,^6^,^7^

The incidence of anthrax in Kenya is not well documented. Anthrax is not included in the Integrated Disease Surveillance and Response strategy for priority diseases. Brucellosis has previously been reported as commonly occurring in pastoralist communities in SSA,^8^ whereas undiagnosed rickettsial infections are prevalent in northeastern and western Kenya.^2^,^9^ Rodents and domestic animals live in close proximity to humans in many SSA countries and may harbor several tick and flea species that are potential vectors of rickettsial pathogens.^2^,^9^ Typically, IgG antibodies for different pathogens persist over a long period, but durability for this persistence is not well characterized. We conducted a study to estimate the seroprevalence of antibodies to *Bacillus anthracis*, *Brucella* spp., spotted fever group rickettsioses (SFGR), and typhus group rickettsioses (TGR) in Kenya and the factors associated with previous exposure to these pathogens.

## Methods

### Sample selection.

Archived serum specimens that had been collected as part of the 2007 Kenya AIDS indicator Survey (KAIS 2007) were used for this study.^10^ The KAIS 2007 was a national population-based cross-sectional study in which serum was collected from persons aged 15–64 years residing in households in all provinces of Kenya. The aim of KAIS 2007 was to provide representative information on the status of human immunodeficiency virus/acquired immunodeficiency syndrome (HIV/AIDS) in Kenya. After providing informed consent, respondents provided information on demographics, risk behaviors, and uptake of HIV interventions and a sample of blood for HIV testing at a central laboratory. Remnant blood samples were stored at −80°C for future unspecified testing. For the purposes of this study, sample sizes needed to establish representative seroprevalence with a precision of 0.025 calculated for each province, taking into account the design effect and intra-cluster correlation (ρ, assumed to be 0.2).^11^ This was calculated for each of the zoonotic bacterial infections. Samples without sufficient sample remaining (including the HIV-positive samples) were removed from the random selection process. Samples with adequate volume were randomly selected using STATA v12.1 (College Station, TX). This was done based on proportional sampling across the eight provinces of Kenya to test for evidence of previous exposure to bacterial zoonotic pathogens including *B. anthracis*, *Brucella* spp., SFGR, and TGR. HIV-positive samples were not included in this study because they were prioritized for use in other studies related to HIV burden.

### Serological testing.

Each serum specimen was first heat inactivated at 56°C for 30 minutes and then tested for IgG-specific antibodies against *B. anthracis*, *Brucella* spp., SFGR, and TGR by enzyme-linked immunosorbent assay (ELISA). Because of the limited volumes, all specimens were diluted to a ratio of 1:1 in phosphate-buffered saline (PBS) to expand sample volume.

We tested specimens for the presence of IgG antibodies against *B. anthracis* using the Abnova Protective Antigen IgG ELISA kit^®^ (Abnova GmbH, Heidelberg, Germany) according to the manufacturer's instructions. In brief, 2.5 μL test sample and controls were diluted 1:41 and added into the pre-coated plates in duplicate followed by incubation at room temperature for 30 minutes. The plates were washed three times with 300 μL of 1× wash buffer. Of the conjugate solution, 100 μL was added to each well and incubated at room temperature for 30 minutes.

The plates were washed again as previously described and 100 μL of 3,3′,5,5′-tetramethylbenzidine (TMB) substrate solution added to each well with 10-minute incubation at room temperature. Finally, a stop solution was added and absorbance (optical density [OD]) for each plate read at 450 nm using the ELx800 absorbance microplate reader (BioTek, VT). The calibrator OD was multiplied by the calibrator factor on each bottle to determine the cutoff value. Each sample and control OD was divided by the cutoff value to determine the antibody index. We considered an antibody index > 1.1 as positive and indicative of exposure to *B. anthracis*, 0.9–1.1 as borderline positive, and < 0.9 as negative.

We tested the serum for presence of IgG antibodies against *Brucella* spp. using the *Brucella* IgG ELISA Kit (Immuno-Biological Laboratories, Minneapolis, MN) according to the manufacturer's instructions. In brief, 5 μL of each heat-inactivated sample was diluted 1:101 and incubated for 1 hour at room temperature. Plates were washed three times with 300 μL of 1× wash buffer. Of anti-human-IgG-HRP (rabbit) conjugate, 100 μL was added into each well and incubated at room temperature for 30 minutes and then washed as mentioned earlier. Subsequently, 100 μL TMB substrate was added to each well and plates were incubated at room temperature for 20 minutes. Then, 0.5 M sulfuric acid stop solution was added to each well and absorbance read at 450 nm using the ELx800 absorbance microplate reader. The test sera and negative control (calibrator A), cutoff standard (calibrator B), weak positive (calibrator C), and positive control (calibrator D) were tested in duplicate and a substrate blank was included.

Samples were compared with a typical calibration curve for the different test calibrators ranging from 1 unit/mL, OD = 0.048 for the negative control (calibrator A) to 150 units/mL, OD = 2.035 for the positive control (calibrator D). Any specimen that had > 12 units/mL were considered as positive for *Brucella* spp., 8–12 units/mL was considered equivocal, and < 8 units/mL was negative.

*Rickettsia* spp. antibodies were detected using antigens obtained from the Viral and Rickettsial Diseases Department, U.S. Naval Medical Research Center, Bethesda, MD. Before testing, these antigens (spotted fever group *Rickettsia Conorii* [VR141] and *Rickettsia typhi* [Wilmington]) were diluted in PBS at 1:1,500 and 1:3,000, respectively. Half of the test plates were coated with 100 μL of the diluted antigen and the other half with PBS and incubated overnight at 4°C. Test plates were then washed three times with 300 μL of wash buffer and blocked with 200 μL of 10% skimmed milk in wash buffer. The plates were then incubated at room temperature for 1 hour and washed as stated above. Five microliters each of the heat-inactivated patient samples and negative and positive controls were then diluted in 5% skimmed milk at 1:100 dilutions and 100 μL of this was loaded onto the test plates in duplicate and incubated at room temperature for 1 hour. Subsequently, the plates were washed six times as mentioned above and 100 μL of peroxidase goat antihuman IgG conjugate added before incubation at room temperature for 1 hour. Plates were washed six times and 100 μL of substrate added with incubation at room temperature in the dark. Absorbance wavelengths were read at 405 nm using the ELx800 absorbance microplate reader. A positive *Rickettsia* spp. sample had a net total absorbance > 0.5 with an adjusted OD > 0.2.

### Statistical methods.

Data were analyzed using survey procedures of Statistical Analysis Software (SAS) version 9.3 (SAS Institute Inc., Cary, NC). The calculation of seroprevalence was adjusted to account for the variation in sampling of the study population. Sampling weights for households, individual interviews, blood draws, and pathogen testing were used to correct for unequal probability of selection and to adjust for nonresponse. These were used to represent the larger Kenyan population from which the samples were drawn. A cluster was required to have a minimum of three samples on sampling and when this was not achieved due to the small sample size of negative HIV blood samples, nonpositive sampling weights were obtained and were therefore excluded from analysis. Estimates were weighted to account for unequal probability of selection and to adjust for nonresponse to produce results that were representative at the national and provincial level from which the sample was drawn.

We estimated the national and provincial seroprevalence of four bacterial zoonotic diseases: *B. anthracis*, *Brucella* spp., SFGR, and TGR and presented estimates as weighted percentages and 95% confidence intervals (CIs). We conducted bivariate analysis to test for potential associations between the bacterial zoonotic diseases and select demographic variables including sex, age, education level, wealth, and province. Variables that were associated with the outcome variable at a significance level of *P* value < 0.1 were included in a multivariate logistic regression model. Variables that remained associated with the outcome at a significance level of *P* value < 0.05 in multivariate analysis were considered to be independently and significantly associated with the outcome. Interaction terms were considered in the multiple logistic regressions for education, wealth, and location of residence to evaluate whether these interactions improved the models. Akaike information criterion (AIC) was used to evaluate the significance of interaction terms on the models, that is, if the AIC in the model with interaction is less than in the model without interaction, then the interaction improved the model. We present unadjusted odds ratios (ORs), adjusted odds ratios (AORs), and 95% CIs as measures of associations. ArcGIS version 10.2 (Redlands, CA) was used to geographically plot the IgG antibody seroprevalence across the eight provinces of Kenya.

The seroprevalence results were linked to the KAIS 2007 database using a unique identifier. The KAIS database included both household- and individual-level information.

### Ethical considerations.

The KAIS 2007 protocol was approved by the Scientific Steering Committee and the Ethical Review Committee at the Kenya Medical Research Institute (protocol no. 1209) and by the Institutional Review Board (IRB) at the U.S. Centers for Disease Control and Prevention under IRB protocol no. 5169.

## Results

**Figure 1.**
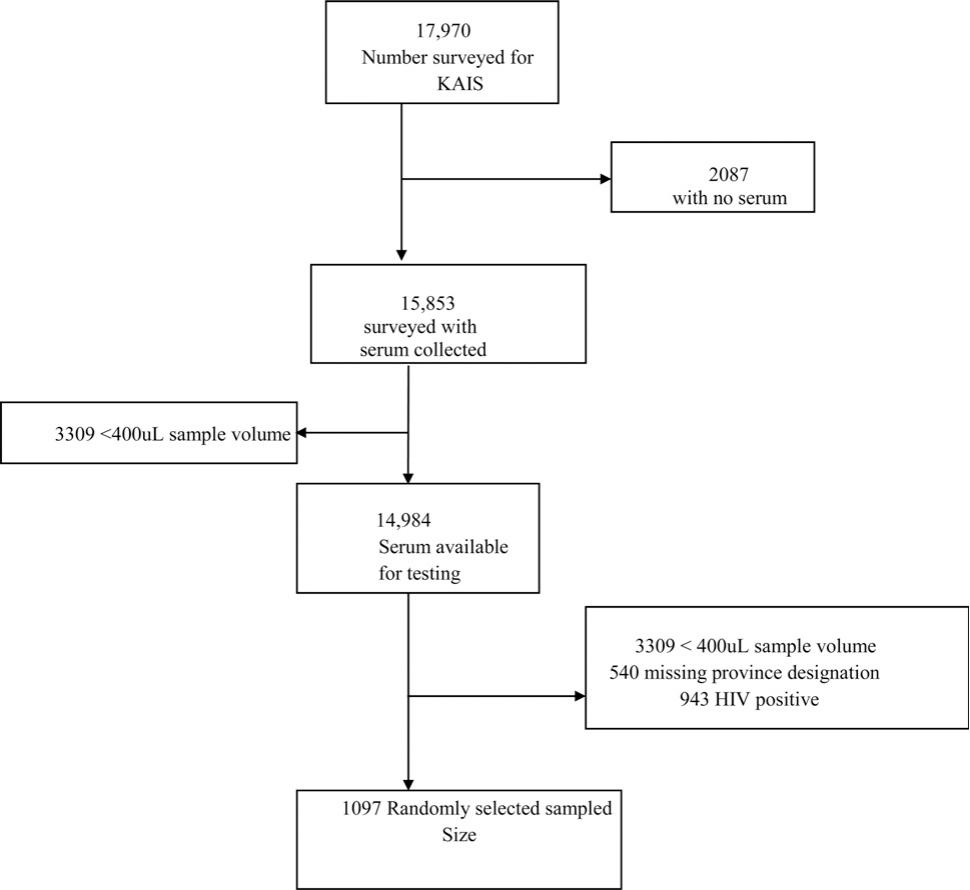
Flow diagram indicating sample selection.

**Figure 2.**
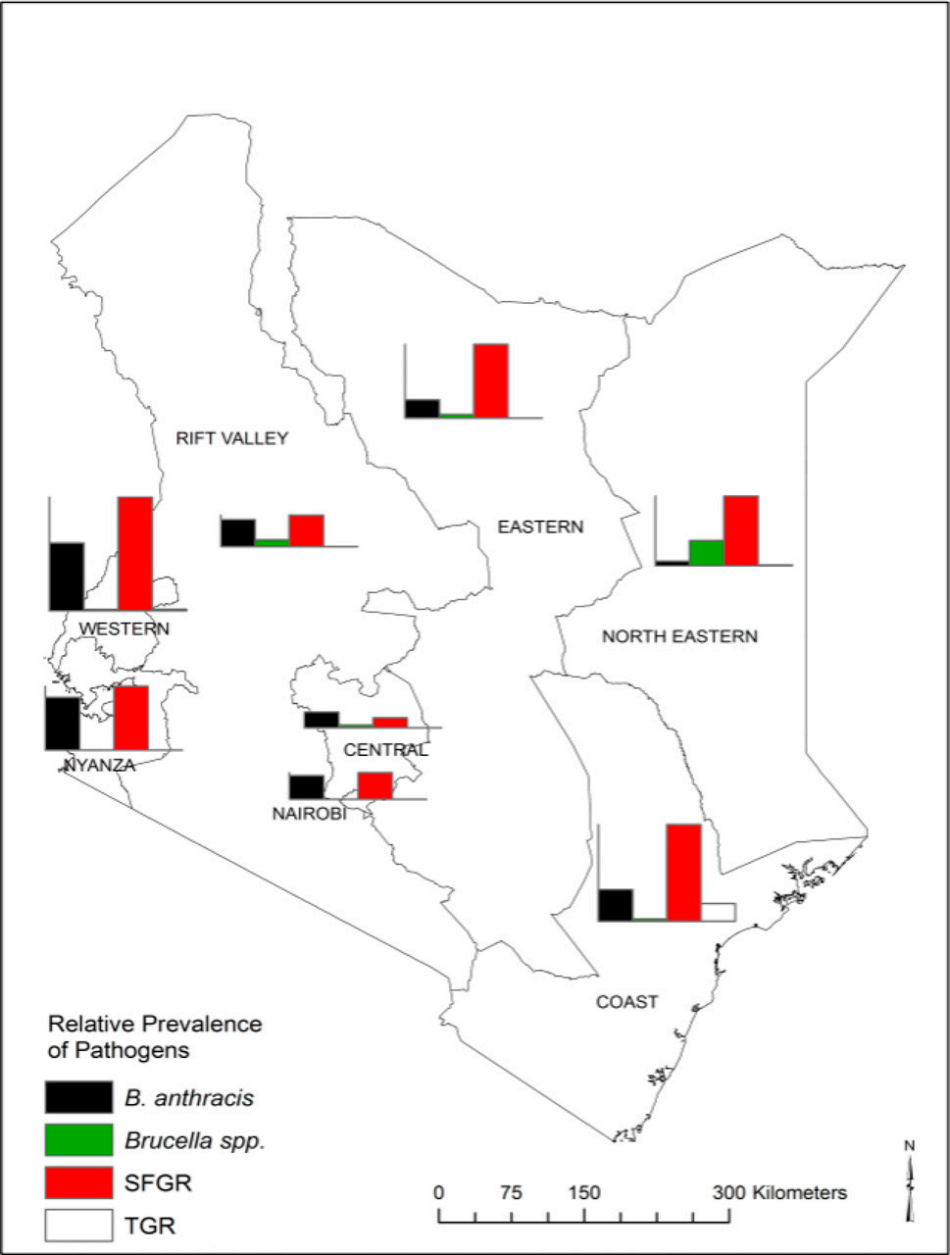
Seroprevalence of to *Bacillus anthracis*, *Brucella* spp., spotted fever group rickettsioses (SFGR), and typhus group rickettsioses (TGR) by province in Kenya, 2007. The bar graphs have the same *y* axis.

A total of 17,970 persons participated in the KAIS 2007 serosurvey, of whom 15,853 (88.4%) had blood draws for HIV testing (Figure 1

). Of these blood draws, 14,984 (94.5%) were stored and available for future testing including 943 HIV-positive and 14,041 HIV-negative samples. A subset of 1,091 (7.8%) serum samples was selected for IgG antibody testing against specific zoonotic bacterial agents. Of the samples, 661 (61%) were from females. The seroprevalence of *B. anthracis* was 11.3% (95% CI = 8.2–14.4%) nationally (range = 1.9% [95% CI = 0.0–4.1%] in North Eastern Province to 27.5% [95% CI = 18.4–36.7%] in Western Province). *Brucella* spp. seroprevalence was 3.0% (95% CI = 1.0–5.0%) nationally (range = 0% in Nairobi and Nyanza provinces to 10.3% [95% CI = 0–21.8%] in North Eastern province). SFGR prevalence was 23.3% (95% CI = 19.4–27.2%) nationally (range = 4.1% [95% CI = 0.2–8.1%] in Central Province to 46.1% [95% CI = 34.0–58.2%] in Western Province). TGR seroprevalence was 0.4% (95% CI = 0.2–1.1%) nationally (range = 0% in Nairobi, Central, Eastern, Nyanza, and Rift Valley provinces to 7.2% [95% CI = 1.1–13.4%] in North Eastern Province) (Figure 2

).

### Factors associated with *B. anthracis*.

In bivariate analysis, province of residence was significantly associated with higher odds of positive titers to *B. anthracis* (*P* < 0.0001): persons living in Western Province had 20-fold higher odds of positive titers to *B. anthracis* than those in North Eastern Province (*P* < 0.0001). Odds of positive titers to *B. anthracis* were also significant for Nairobi, Coast, Nyanza, and Rift Valley provinces (Table 1). Persons in the second and fourth wealth quintiles had more than three times higher odds of positive titers to *B. anthracis* than those in the highest wealth quintile (*P* = 0.016 and 0.018, respectively). Although education level was not significantly associated with positive titers to *B. anthracis*, persons who had some primary education had up to 2.3 times higher odds of having positive titers to *B. anthracis* than those with no primary education.

After adjusting for residence, sex, age, education level, and wealth in multivariate analysis, all the provinces of Kenya were significantly associated with higher odds of positive titers to *B. anthracis* with AOR ranging from 5.83 (95% CI = 1.24–27.35) for Eastern Province to 25.41 (95% CI = 6.4–100.92) for Western Province. Wealth quintiles remained significantly and independently associated with higher odds of positive titers to *B. anthracis*. The poorest wealth quintile gained significance after adjustment for other demographic factors. Education level was not significantly and independently associated with higher odds of positive titers to *B. anthracis* after adjustment for sex, age, wealth, and province in multivariate analysis. Residence was not significant (*P* = 0.902) and therefore not included in the interaction terms for the model. The model increased from AIC = 782 (without interaction terms) to AIC = 784 (with interaction terms), which included education level and wealth, and therefore, the interaction terms were not included in the model.

### Factors associated with *Brucella* spp.

In bivariate analysis, we found statistically significant associations between positive titers to *Brucella* spp. with residence, sex, education level, and wealth (*P* < 0.05) (Table 2). Those residing in rural areas had up to 14 times higher odds of having positive titers to *Brucella* spp. than those residing in urban areas (*P* = 0.0133). Males, persons aged 50–64 years, and those with no primary school education were 3- to 4-fold more likely to have positive titers to *Brucella* spp. (*P* < 0.05) than females, persons aged 30–49 years, and those with some primary school education, respectively. Persons within the lowest wealth quintile had up to 50 times higher odds of having positive titers to *Brucella* spp. than those in the highest wealth quintile (*P* = 0.0003). Persons living in North Eastern Province had up to 21 times higher odds of having positive titers to *Brucella* spp. than those in Nyanza Province (*P* = 0.0113). In multivariate analysis that controlled for residence, wealth, and province, males, persons aged 15–29 and 50–64 years and those with no primary education had significant higher odds of having positive titers to *Brucella* spp. Despite residence (*P* = 0.013), education level/wealth (*P* < 0.001) and residence/education level (*P* < 0.0001) being significant on bivariate analysis; they did not improve the model, that is, AIC = 42.2 (without interaction) and AIC = 49.9 (with interaction).

### Factors associated with SFGR.

In bivariate analysis, residence, age, education level, wealth, and province of residence were statistically associated with positive titers to SFGR (*P* < 0.05). Those with no primary school education or some primary education had higher odds of having positive titers to SFGR than those with complete primary or secondary education (Table 3). There was significant difference in the wealth categories with those in the lowest, second, and middle quintiles having a higher odds of having positive titers to SFGR than those in the highest wealth quintile. Persons living in Western Province of Kenya had up to 20 times higher odds of having positive titers to SFGR than those living in Central Province (OR = 19.9, *P* < 0.0001). On multivariate analysis controlling for residence, age, education level and wealth, persons living in Western and Coast provinces had 13- to 21-fold higher odds of having positive titers to SFGR than those in Central Province. There was a significant association between having positive titers to SFGR and being male (OR = 1.8, *P* = 0.03). Although the interaction terms for residence/education (*P* = 0.129) and wealth/education (*P* = 0.5418) levels were not significant, they improved the model from AIC = 91.969 (without interaction) to AIC = 87.643 (with interaction). Those who resided in rural areas and had incomplete primary education had up to 2.7 times higher odds of having positive titers for SFGR than those from urban areas who had completed primary or secondary education (Table 4).

### Factors associated with TGR.

In bivariate analysis, there was no significant association between positive titers to TGR with residence, sex, age, education level, or wealth (Table 5). However, those in the lowest wealth quintile were significantly associated with positive TGR titers compared with those in the second wealth quintile (OR = 7.35, *P* = 0.041). Province was significantly associated with positive titers to TGR (*P* < 0.05). Persons residing in Coast Province were significantly associated with having positive titers to TGR than those residing in North Eastern Province of Kenya (*P* < 0.05). There were no other significant factors in the bivariate analysis, and therefore logistic multivariate regression was not done for TGR.

## Discussion

Many infectious diseases go undetected in SSA because of nonspecific signs and symptoms of fever, poor access to health-care systems, limited knowledge by health practitioners, and challenges in diagnostics.^1^–^4^ This study provides baseline estimates of national and provincial seroprevalence of select zoonotic bacterial diseases in Kenya. Our study complemented the findings of previous studies in Kenya that showed evidence of acute infections in humans caused by these pathogens.^2^,^9^

Evidence of positive titers to *B. anthracis* was detected predominantly within the western region of Kenya. The odds of positive titers to *B. anthracis* were highest in Nairobi, Coast, Nyanza, Rift Valley, and Western provinces compared with North Eastern Province. This requires further analytical studies for a comprehensive understanding with focus on soil conditions. It has also been shown that animal husbandry practices including mixed farming of herds and ruminants in Kenya increase the risk of bacterial zoonotic infections.^17^ In Nyanza Province, it has been reported that up to 70% of the public health workers have encountered zoonotic diseases at work, especially rabies, anthrax, and brucellosis.^18^ This is similar to our findings, where participants in Nyanza Province had higher odds of positive titers to *B. anthracis* compared with participants in North Eastern Province. Previous studies have shown that participants with limited formal education were more likely to have zoonotic infections.^7^ Health practitioners in rural areas have been found to have limited knowledge of bacterial zoonotic diseases leading to underdiagnosis and undertreatment, further compounding the morbidity from these diseases.^7^ In contrast, this study shows that participants with no primary school education had low positive titers to *B. anthracis* unlike the other select bacterial zoonotic diseases. However, the colinearity between primary education and province of residency was not evaluated. Participants who are below the fourth wealth quintile had higher odds of having positive titers to *B. anthracis* as compared with those in the highest wealth quintile. Interaction for education level and wealth did not improve the model for evidence of exposure to *B. anthracis.*

In this study, positive titers to *Brucella* spp. were lowest in Nyanza Province but predominant in North Eastern Province where livelihoods are heavily dependent on camel and other livestock breeding and commercial enterprises consistent with previous studies that detected the antibodies in pastoral communities of Kenya.^12^ Residents in the rural areas, males, and participants living in North Eastern Province had higher odds of having positive titers to *Brucella* spp., possibly due to the male predominance among livestock workers especially in rural areas.^12^ In rural pastoral communities, males have more animal contact including skinning, slaughtering, sleeping with herd animals, and caring for animals during birthing and are therefore highly exposed.^14^ Consumption of unpasteurized milk has also been shown to expose individuals to *Brucella* spp^15^ however, we have no information suggesting that unpasteurized milk consumption is more prevalent in North Eastern Province. Older participants aged 50–64 years had significantly higher odds of having positive titers to *Brucella* spp. This finding may simply be a feature of increased potential of exposure over an added number of years combined with sustained antibody levels after exposure. Participants who are below the middle wealth quintile had higher odds of having positive titers to *Brucella* spp. as compared with those in the highest wealth quintile. Interactions terms including education level/wealth and residence/education level did not improve the model fit for exposure to *Brucella* spp.

Positive titers to SFGR was detected predominantly within the western region of Kenya as reported in previous studies.^18^ Participants who are below the middle wealth quintile had higher odds of having positive titers to SFGR as compared with those in the highest wealth quintile. Previous studies have shown a close relationship between infectious diseases and socioeconomic factors.^16^ However, we found that the odds of having positive titers to TGR were higher in those with a high socioeconomic status. This might be due to the high socioeconomic status of the livestock-dominated pastoralists of North Eastern Province who trade in slaughter of cows and camels. Similarly, other studies have shown a close association between increased herd sizes with occurrence of zoonotic diseases in SSA.^12^ However, increased herd size increases the exposure to vectors including ticks and fleas, and therefore, the socioeconomic status may be a confounder. There appears to be limited exposure to TGR in Kenya compared with SFGR as previously shown.^9^,^13^

Interaction terms including residence/education level and wealth/education level improved the model for SFGR. There were no significant factors to be included as interaction terms for TGR.

## Limitation

Since this was a cross-sectional serological prevalence survey of samples collected in 2007, our study could not reveal the direction of the observed association. We had a limited sample size, resulting in wide CIs around the point estimates and measures of association. The durability for IgG antibodies was not evaluated. HIV-positive specimens were not tested for these select agents, which could have potentially biased our results. The data set was limited to residence (rural and urban), age, education level, and income and did not include data about activities known to put people at increased risk such as soil conditions, slaughtering animals, handling aborted animals, milking animals, consumption of raw milk, and eating infected meat.

## Conclusion

National serological surveys can be leveraged to provide important information on burden of zoonotic pathogens to identify areas at risk and ultimately to measure impact of interventions. Evidence of positive antibody titers against *B. anthracis* and SFGR were common in Western Province, whereas positive antibody titers against *Brucella* spp. was common in North Eastern Province; positive TGR titer was less common across the country. Our findings suggest that some bacterial zoonotic diseases are widespread across the country and should therefore be considered in the differential diagnosis of febrile patients in Kenya. This study maps the geographical distribution of bacterial zoonotic infections by province and indicates areas for targeted public health interventions in Kenya such as enhancing surveillance, encouraging hygienic slaughter practices, culling affected animals, and pasteurizing milk. Better appreciation of the disease burden of these diseases might lead to better diagnostic capacity and improved clinical recognition and case management. Introduction of rapid diagnostic tests might facilitate earlier detection of bacterial zoonotic diseases in the country.

## Figures and Tables

**Table 1:** Bivariate and multivariate analysis of factors associated with previous exposure to *Bacillus anthracis*

Variable	Positive (*n*)	Total (*N*)	% Positive (95% CI)	Unadjusted OR (95% CI)	*P* value	G *P* value	AOR (95% CI)	M *P* value
Residence								
Rural	110	784	11.2 (7.8–14.6)	1.0 (0.5–2.0)	0.902	0.902	–	–
Urban	31	307	11.6 (5.2–18.1)	REF	–	–	REF	–
Sex								
Male	61	430	13.6 (7.8–19.4)	1.4 (0.8–2.5)	0.2139	0.2139	–	–
Female	80	661	9.9 (6.8–13.0)	REF	–	–	REF	–
Age (in years)								
15–29	67	529	11.0 (7.5–14.6)	REF	–	–	REF	–
30–49	54	395	11.4 (5.4–17.4)	1.0 (0.5–2.0)	0.9228	–	–	–
50–64	20	167	12.2 (5.8–18.5)	1.12 (0.6–2.2)	0.7571	0.9532	–	–
Education level								
No primary	22	223	7.0 (2.4–11.6)	REF	–	–	REF	–
Some primary	47	295	14.5 (10.1–18.9)	**2.3** **(1.07**–**4.76)**	0.0322*	0.1007	0.91 (0.5–1.67)	0.7693
Complete primary and secondary+	72	573	11.9 (7.7–16.1)	1.8 (0.9–3.8)	0.1236	–	1.16 (0.56–2.4)	0.6883
Wealth quintiles								
Lowest	33	234	10.7 (5.9–15.6)	1.99 (0.85–4.66)	0.1145	–	**4.91 (1.57**–**15.4)**	0.0063*
Second	44	189	17.1 (7.0–27.2)	**3.42 (1.26**–**9.28)**	0.016*	0.0622	**5.57 (1.77**–**17.51)**	0.0033*
Middle	23	188	9.1 (5.1–13.0)	1.65 (0.71–3.83)	0.2466	–	2.0 (0.64–6.19)	0.2312
Fourth	22	186	18.5 (6.8–30.3)	**3.77 (1.26**–**11.30)**	0.018*	–	**4.56 (1.15**–**18.14)**	0.0313*
Highest	19	294	5.7 (2.0–9.4)	REF	–	–	REF	–
Province								
Nairobi	9	153	9.5 (1.5–17.6)	**5.46 (1.20**–**24.88)**	0.0281*	–	**18.27 (2.84**–**117.39)**	0.0022*
Central	10	150	6.4 (2.5–10.3)	3.55 (0.91–13.86)	0.0679	–	**6.48 (1.34**–**31.36)**	0.0203*
Coast	20	129	12.8 (6.1–19.5)	**7.63 (2.01**–**29.04)**	0.0029*	–	**13.39 (3.09**–**57.9)**	0.0005*
Eastern	11	112	7.3 (2.0–12.7)	4.11 (0.98–17.24)	0.0531	–	**5.83 (1.24**–**27.35)**	0.0253*
North Eastern	7	140	1.9 (0.0–4.1)	REF	–	–	REF	–
Nyanza	35	154	21.4 (13.3–29.4)	**14.09 (3.88**–**51.14)**	< 0.0001*	–	**18.19 (4.41**–**74.91)**	< 0.0001*
Rift Valley	10	117	11.0 (1.3–20.8)	**6.44 (1.36**–**30.48)**	0.019*	–	**10.55 (2.32**–**48.03)**	0.0023*
Western	39	136	27.5 (18.4–36.7)	**19.71 (5.49–70.84)**	< 0.0001*	< 0.0001*	**25.41 (6.4–100.92)**	< 0.0001*

AOR = adjusted odds ratio; CI = confidence interval; REF = reference.

*P* value is the category *P* value, G *P* value is the global *P* value for the bivariate variable, M *P* value is the category multivariate *P* value.

* Bold values have significant *P* values.

**Table 2:** Bivariate and multivariate analysis of factors associated with previous exposure to *Brucella* spp.

Variable	Positive (*n*)	Total (*N*)	% Positive (95% CI)	Unadjusted OR (95% CI)	*P* value	G *P* value	AOR (95% CI)	M *P* value
Residence								
Rural	26	694	4.2 (1.2–7.1)	**14.20 (1.74–116.18)**	0.0133*	0.0133*	0.48 (0.0–5.5)	0.5555
Urban	1	269	0.3 (0.0–0.9)	REF	–	–	REF	–
Sex								
Male	17	374	5.3 (1.7–8.9)	**3.49 (1.83–6.63)**	0.0001*	0.0001*	**4.67 (2.37–9.19)**	< 0.0001*
Female	10	589	1.6 (0.2–2.9)	REF	–	–	REF	–
Age (in years)								
15–29	11	461	2.9 (0.8–5.1)	1.52 (0.7–3.31)	0.2901	–	**5.09 (1.53–17.00)**	0.0081*
30–49	9	353	2.0 (0.3–3.6)	REF	–	–	REF	–
50–64	7	149	6.3 (0.3–12.3)	**3.37 (1.15–9.86)**	0.0265*	0.0853	**3.38 (1.08–10.65)**	0.0371*
Education level								
No primary	22	202	9.8 (1.4–18.3)	**15.76 (3.91–63.54)**	0.0001*	0.0001*	**7.29 (1.48–35.94)**	0.0146*
Some primary	5	259	0.7 (0. –1.4)	REF	–	–	REF	–
Complete primary and secondary+	–	–	–	–	–	–	–	–
Wealth quintiles								
Lowest	18	216	10.0 (2.6–17.3)	**50.1 (5.91–425.07)**	0.0003*	–	12.78 (0.74–219.83)	0.0793
Second	3	166	1.6 (0.0–3.9)	7.52 (0.65–87.22)	0.1067	< 0.0001*	2.34 (0.11–48.15)	0.5807
Middle	5	160	2.8 (0.3–5.3)	**13.1 (1.51–113.51)**	0.0196*	–	8.38 (0.48–147.27)	0.1461
Fourth	–	–	–	–	–	–	–	–
Highest	1	256	0.2 (0.0–0.7)	REF	–	–	REF	–
Province								
Nairobi	–	–	–	–	–	–	–	–
Central	2	141	1.1 (0.0–2.7)	2.06 (0.18–23.97)	0.5628	–	5.51 (0.50–61.12)	0.1644
Coast	1	113	1.0 (0.0–2.9)	1.79 (0.11–29.77)	0.6853	–	1.37 (0.06–30.47)	0.8438
Eastern	1	96	1.5 (0.0–4.3)	2.72 (0.17–44.68)	0.4833	–	2.57 (0.25–25.96)	0.4249
North Eastern	20	129	10.3 (0.0–21.8)	**20.86 (1.99–218.72)**	0.0113*	–	5.52 (0.50–61.27)	0.1639
Nyanza	–	–	–	REF	–	–	–	–
Rift Valley	2	106	2.8 (0.0–7.0)	5.29 (0.43–64.39)	0.1916	0.0679	2.97 (0.27–32.00)	0.3705
Western	1	126	0.5 (0.0–1.6)	REF	–	–	REF	–

AOR = adjusted odds ratio; CI = confidence interval; REF = reference.

*P* value is the category *P* value, G *P* value is the global *P* value for the bivariate variable, M *P* value is the category multivariate *P* value.

* Bold values have significant *P* values.

**Table 3:** Bivariate and multivariate analysis of factors associated with previous exposure to SFGR

Variable	Positive (*n*)	Total (*N*)	% Positive (95% CI)	Unadjusted OR (95% CI)	*P* value	G *P* value	Adjusted OR (95% CI)	M *P* value
Residence								
Rural	160	558	26.7 (22.3–31.1)	**2.29 (1.23–4.24)**	0.0088*	0.0088*	2.33 (0.80–6.77)	0.1211
Urban	31	212	13.8 (6.9–20.6)	REF	–	–	REF	–
Sex								
Male	91	317	27.2 (21.5–32.8)	1.45 (0.94–2.23)	0.0937	0.0937	1.80 (1.05–3.06)	0.0308*
Female	100	453	20.5 (15.3–25.7)	REF	–	–	REF	–
Age (in years)								
15–29	88	368	22.7 (17.0–28.3)	1.24 (0.85–1.8)	0.2703	–	1.47 (0.93–2.33)	0.1024
30–49	63	285	19.1 (14.9–23.4)	REF	–	–	REF	–
50–64	40	117	37.7 (19.2–56.2)	**2.56 (1.15–5.71)**	0.0217*	0.0361*	1.97 (0.81–4.80)	0.1364
Education level								
No primary	59	156	36.5 (28.6–44.4)	**3.56 (2.09–6.08)**	< 0.0001*	< 0.0001*	0.66 (0.15–2.94)	0.5866
Some primary	60	211	27.4 (19.7–35.1)	**2.34 (1.42–3.87)**	0.0009*	–	8.40 (0.39–182.39)	0.1753
Complete primary and secondary+	72	403	13.9 (9.6–18.2)	REF	–	–	REF	–
Wealth quintiles								
Lowest	53	161	30.9 (23.1–38.8)	**3.24 (1.74–6.03)**	0.0002*	–	0.95 (0.27–3.32)	0.9398
Second	52	147	31.4 (23.8–38.9)	**3.31 (1.81–6.07)**	0.0001*	0.0013*	0.58 (0.16–2.09)	0.4077
Middle	34	130	23.1 (15.1–31.1)	**2.18 (1.16–4.09)**	0.0155*	–	0.98 (0.37–2.61)	0.962
Fourth	25	129	18.2 (8.9–27.4)	1.61 (0.79–3.3)	0.1926	–	0.68 (0.30–1.57)	0.3659
Highest	27	203	12.1 (6.8–17.5)	REF	–	–	REF	–
Province								
Nairobi	11	110	10.8 (2.1–19.5)	2.82 (0.73–10.88)	0.1325	–	**6.84 (1.19–39.27)**	0.0309*
Central	4	106	4.1 (0.2–8.1)	REF	–	–	**REF**	–
Coast	32	89	39.2 (23.0–55.3)	**15.00 (4.48–50.21)**	< 0.0001*	–	**13.75 (3.80–49.65)**	< 0.0001*
Eastern	22	77	29.9 (15.5–44.3)	**9.92 (2.95–33.41)**	0.0002*	–	**10.51 (2.85–38.75)**	0.0004*
North Eastern	29	100	28.2 (21.8–34.6)	**9.14 (3.20–26.14)**	< 0.0001*	–	**5.10 (1.41–18.51)**	0.0131*
Nyanza	34	106	25.8 (13.0–38.7)	**8.11 (2.42–27.03)**	0.0007*	–	**8.90 (2.46–32.22)**	0.0009*
Rift Valley	12	83	12.8 (1.3–24.2)	3.41 (0.81–14.33	0.0938	–	2.99 (0.68–13.10)	0.147
Western	47	99	46.1 (34.0–58.2)	**19.9 (6.54–60.58)**	< 0.0001*	< 0.0001*	**21.13 (6.03–74.00)**	< 0.0001*

AOR = adjusted odds ratio; CI = confidence interval; SFGR = spotted fever group rickettsioses; REF = reference.

*P* value is the category *P* value, G *P* value is the global *P* value for the bivariate variable, M *P* value is the category multivariate *P* value.

* Bold values have significant *P* values.

**Table 4:** Interaction terms fitted into the SFGR model

Variable	AOR (95% CI)	M *P* value
Residence by education level		
Rural/no primary	0.35 (0.025–5.01)	0.4399
Urban/complete primary and secondary+	REF	–
Education level by wealth quintiles		
Lowest/incomplete primary	0.63 (0.11–3.67)	0.606
Second/incomplete primary	1.55 (0.29–8.31)	0.6097
Middle/incomplete primary	1.02 (0.20–5.13)	0.9821
Fourth/incomplete primary	1.19 (0.26–5.39)	0.826
Lowest/no primary	0.81 (0.02–27.08)	0.9083
Second/no primary	2.14 (0.06–74.15)	0.6736
Middle/no primary	0.48 (0.01–17.92)	0.6888
Fourth/no primary	1.55 (0.14–17.35)	0.7241
Highest/complete primary and secondary+	REF	REF

AOR = adjusted odds ratio; CI = confidence interval; SFGR = spotted fever group rickettsioses; REF = reference.

M *P* value is the category multivariate *P* value.

**Table 5:** Bivariate and multivariate analysis of factors associated with previous exposure to TGR

Variable	Positive (*n*)	Total (*N*)	% Positive (95% CI)	Unadjusted OR (95% CI)	*P* value	G *P* value	AOR (95% CI)	M *P* value
Residence								
Rural	8	558	0.5 (0.1–1.0)	0.5541 (0.1256–2.4452)	0.4357	0.4357	–	–
Urban	4	212	1.0 (0.0–2.2)	REF	–	–	REF	–
Sex								
Male	3	317	0.5 (0.0–1.1)	REF	–	–	REF	–
Female	9	453	0.8 (0.3–1.4)	1.73 (0.48–6.29)	0.4045	0.4045	–	–
Age (in years)								
15–29	5	368	0.7 (0.2–1.2)	1.93 (0.56–6.72)	0.3002	–	–	–
30–49	3	285	0.4 (0.0–0.8)	REF	–	–	REF	–
50–64	4	117	1.5 (0.0–3.4)	4.12 (0.81–20.99)	0.0886	0.229	–	–
Education level								
No primary	5	156	0.9 (0.0–2.0)	1.76 (0.34–9.12)	0.5028	0.794	–	–
Some primary	3	211	0.7 (0.0–1.5)	1.38 (0.25–7.57)	0.7132	–	–	–
Complete primary and secondary+	4	403	0.5 (0.0–1.2)	REF	–	–	REF	–
Wealth quintiles								
Lowest	5	161	1.2 (0.1–2.3)	**7.35 (1.09–49.75)**	0.041*	–	–	–
Second	1	147	0.2 (0.0–0.5)	REF	–	–	REF	–
Middle	1	130	0.3 (0.0–0.8)	1.54 (0.09–27.19)	0.7663	0.1108	–	–
Fourth	4	129	1.3 (0.0–3.1)	8.21 (0.69–97.30)	0.0952	–	–	–
Highest	1	203	0.4 (0.0–1.2)	2.46 (0.14–42.98)	0.5382	–	–	–
Province								
Nairobi	–	–	–	–	–	–	–	–
Central	–	–	–	–	–	–	–	–
Coast	10	89	7.2 (1.1–13.4)	**23.16 (2.14**–**250.24)**	0.0097*	–	–	–
Eastern	–	–	–	–	–	–	–	–
North Eastern	1	100	0.3 (0.0–1.1)	REF	–	–	REF	–
Nyanza	–	–	–	–	–	–	–	–
Rift Valley	–	–	–	–	–	–	–	–
Western	1	99	0.7 (0.0–2.0)	1.94 (0.10–37.96)	0.6601	0.0064*	–	–

AOR = adjusted odds ratio; CI = confidence interval; TGR = typhus group rickettsioses; REF = reference.

*P* value is the category *P* value, G *P* value is the global *P* value for the bivariate variable, M *P* value is the category multivariate *P* value.

* Bold values have significant *P* values.
